# Does the company's economic performance affect access to occupational health services?

**DOI:** 10.1186/1472-6963-9-156

**Published:** 2009-09-02

**Authors:** Eila Kankaanpää, Aki Suhonen, Hannu Valtonen

**Affiliations:** 1 Finnish Institute of Occupational Health, Research and development in OHS, POB 93, FIN-70701 Kuopio, Finland; 2 University of Kuopio, Department of Health Policy and Management, Kuopio, Finland

## Abstract

**Background:**

In Finland like in many other countries, employers are legally obliged to organize occupational health services (OHS) for their employees. Because employers bear the costs of OHS it could be that in spite of the legal requirement OHS expenditure is more determined by economic performance of the company than by law. Therefore, we explored whether economic performance was associated with the companies' expenditure on occupational health services.

**Methods:**

We used a prospective design to predict expenditure on OHS in 2001 by a company's economic performance in 1999. Data were provided by Statistics Finland and expressed by key indicators for profitability, solidity and liquidity and by the Social Insurance Institution as employers' reimbursement applications for OHS costs. The data could be linked at the company level. Regression analysis was used to study associations adjusted for various confounders.

**Results:**

Nineteen percent of the companies (N = 6 155) did not apply for reimbursement of OHS costs in 2001. The profitability of the company represented by operating margin in 1999 and adjusted for type of industry was not significantly related to the company's probability to apply for reimbursement of the costs in 2001 (OR = 1.00, 95%CI: 0.99 to 1.01). Profitability measured as operating profit in 1999 and adjusted for type of industry was not significantly related to costs for curative medical services (Beta -0.001, 95%CI: -0.00 to 0.11) nor to OHS cost of prevention in 2001 (Beta -0.001, 95%CI: -0.00 to 0.00).

**Conclusion:**

We did not find a relation between the company's economic performance and expenditure on OHS in Finland. We suppose that this is due to legislation obliging employers to provide OHS and the reimbursement system both being strong incentives for employers.

## Background

Occupational health services (OHS) are regarded as a fundamental right of every worker. In order to reach the highest attainable standard of health, workers' health at work should be protected [1,2]. Therefore, employers in Finland and in many other countries are obliged by legislation to organize OHS [3]. Because employers cover the costs of OHS it could be that in spite of this requirement OHS expenditure is more determined by economic performance of the company than by law.

Filer and Golbe [4] have described how company's investment in workplace safety is connected to company's economic performance. In general, a company's financial structure substantially affects its real operating decisions and the amount of risk the company is willing to bear, which have an impact on firm's input choices. Both safety and occupational health services are such inputs for a company.

In making decisions on OHS investments the company is balancing the costs and benefits of OHS. Preventive services are supposed to lead to lower occupational accidents and diseases, lower sickness absence and disability pensions which all improve the economic performance of the company [5-7]. Curative medical services within OHS have similar objectives. In addition, curative medical services can be regarded as fringe benefits, that is, the employer offers employees health services (or health insurance) in place of or as an additional monetary wage [8]. Offering generous curative medical services would then lead to employees' lower wage demands.

Investments in safety and health compete with other investments in the company. While companies make decisions on resource allocation the economic situation of the company might affect OHS differently than other input decisions. Acquiring outside funding for OHS investments will be difficult and therefore investment decisions on OHS are dependent on the liquidity of the company (cash flows). Cash flows indicate if there is internal funding available in general, also for investments in OHS.

Filer and Golbe [4] studied investments in safety which also includes investments in capital goods, like equipment. They summarize various models and conclude that the impact indebtedness has on safety investments is ambiguous, mainly due to the capital nature of safety investments. In our study, the costs for occupational health services include only the payments for the OHS providers that will be paid immediately, and the benefits of good OHS will be received in the future. Therefore, we expect that high leverage and the risk of bankruptcy will lower the investment in occupational health services. This is due to share owners' and bondholders' conflicting interests. Owners bear the costs of OHS, in case of bankruptcy the bondholders become the owners of the company and will receive the fruits of OHS, or the costs of neglect.

There is some evidence to support these assumptions but there are only few studies. Nickell and Nicolitsas found in their study that declining company finances lead to lower pay and to lower safety levels as indicated by abolishment of "restrictive practices" such as restrictions to hours of work, manning ratios on machines, and inflexibility of working practices [9]. Abolishment of these restrictions can be interpreted as lowering of safety levels. Filer and Golbe also observed that, in a broad range of industries, the level of safety in a workplace was related to the firm's operating margin and indebtedness [4]. Particularly at low levels of operating margin, firms doing worse also invested less in safety.

The small amount of research in this field may be due to a lack of data. In Finland, however, it is possible to study the relationship between economic performance and OHS expenditure because employers are entitled to reimbursement for about half of the costs of preventive and curative medical occupational health services. Based on the reimbursement claims, the National Social Insurance Institution keeps an employer-based register of the contents and costs of OHS. In addition, Statistics Finland keeps a register with the firms' annual financial statements. These financial statements allow the calculation of key ratios that measure a company's economic performance. Firm specific identification codes made it possible to combine the information in both registers at the firm level.

Based on these administrative sources, we studied if key ratios for a company's economic performance were associated with the OHS expenditure two years later.

## Methods

### Occupational health services

It has been obligatory for employers to organize preventive occupational health services for their employees since 1979. According to a population survey conducted in 2006, two out of three employees had attended an occupational health examination in the past three years, and around half of them had had occupational health personnel assessing their workplace in the past three years. Although organizing curative medical services is voluntary for employers, over 90% of employees can obtain GP level services from their OHS unit. Around half of the primary care level GP visits of these employees take place within OHS [10]. A more detailed description of the Finnish OHS can be found in [3,11]. At the moment, OHS is the only health care system in Finland that provides curative medical services for users without out-of-pocket payments. Therefore, the curative medical services can be regarded as fringe benefits.

Finland introduced public health insurance to reimburse the costs of curative medical care in the private sector in 1964. Since then employers have got reimbursement for the costs of OHS. Employers first pay all costs of OHS and apply for reimbursement within six months after closing their accounts. The share of the costs reimbursed has varied during the over 40 years of reimbursement. In 2001 and still nowadays, the reimbursement is 60% for preventive and 50% for curative medical services. So, despite of the reimbursement the firms will always bear a considerable part of the cost themselves.

### Study design

In a prospective design, data for economic performance of companies from 1999 were used to predict their expenditure on OHS in 2001.

Statistics Finland collects the financial statements of all Finnish firms from the tax authorities. The register also contains data such as the number of persons employed by the company, number of blue- and white-collar workers, year of establishment, registered office, and industry.

The Social Insurance Institution (SII) registers employers' reimbursement applications for OHS. This register contains data on service mix and costs. We chose to use registers from the years 1999 and 2001. In 1999, the renewed reimbursement system where the costs for prevention and curative medical services are reported separately had been in force for four years. As this project was launched, 2001 was the last year for which all reimbursement applications had been processed. Companies apply for reimbursement within six months of closing their accounts. After this, it takes over a year to process all the applications at the SII.

The Social Insurance Institution register was merged with the Statistics Finland data at the Statistics Finland by using firm-specific identification codes. To protect the privacy of the companies we had at our disposal only the merged unidentifiable data.

### The companies

**Figure 1:**
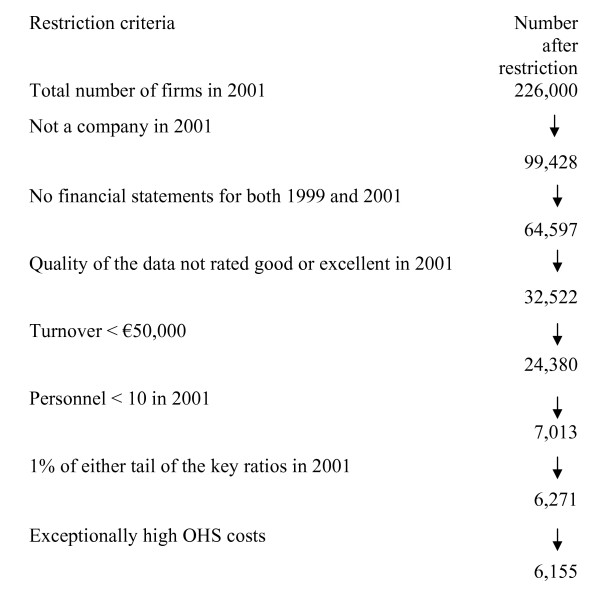
**Construction of the study sample**.

Firms are a heterogeneous group, e.g. differences in the legal construction affect the regulations about bookkeeping. To be sure that the firms in the study would employ personnel and would therefore be obliged to provide OHS we had to restrict our sample (Figure 1). For this reason we excluded firms that

- were not companies

- had not been in business continually through 1999-2001

- had financial statements of insufficient quality as assessed by Statistics Finland (quality is rated low when the firm had not reported many rows in the profit and loss account and this missing information has to be estimated by Statistics Finland)

- had a turnover of less than €50,000 per annum

- had less than ten employees in 2001

- had values in either 1% tail of any key ratio. (Trimming is commonly used with key ratios to help to comply with the normality assumption [12].)

- had very high values of prevention costs (> 240 euro per employee, 61 cases) and curative medical service costs (> 360 euro, 55 cases) in 2001. This indicates that expenditure has been beyond the normal limits because of exceptional circumstances.

This resulted in 6,155 firms that had valid data for both economic performance and OHS expenditure and that could be included in the analysis.

### Economic Performance

Key ratios for profitability are calculated from the profit and loss account by deducting costs from turnover and dividing this margin by turnover. The difference between the ratios results from the variation in costs deducted. The three first key ratios for solidity proportionate the annual profit to different capital titles. The two other key ratios for solidity, relative indebtedness and equity ratio, give an idea about the accumulated wealth of the company. Key ratios for liquidity indicate how large a share of its debts the company could pay with its liquid assets. Definitions for key ratios can be found in the appendix 1 (Table5.

### Statistical analysis

The association between a company's economic performance and investment in OHS was analyzed using regression analysis. The independent variable was economic performance of the company as represented by key indicators for profitability, solidity and liquidity. All indicators were each in its turn modelled and analysed.

Because almost one in five companies had not applied for reimbursement, we first used logistic regression analysis with a dichotomous dependent variable of yes or no spending on OHS. The dependent variable of spending on OHS was constructed from yes or no applying for reimbursement. In a separate analysis we applied linear regression analysis to assess the influence of a company's economic performance on the amount of spending, separately for prevention and curative medical services.

We tested the models using the regression specification error test (RESET test). It can be used for testing the functional form of a model, especially to detect nonlinearities and omitted variables [13]. The test revealed that the relationship between dependent and some independent variables was logarithmic rather than linear. Therefore, these variables were log transformed. The models were also tested for multicollinearity, and there was no multicollinearity.

We assumed that industry, the proportion of blue collar workers, age of the company, geographical region and grade of urbanisation of the area could all be related to the economic performance and to the expenditure of OHS. These variables were therefore introduced as confounders in all models.

We used the STATA software packages for the analysis.

## Results

### Study Sample

The companies included in the study were a representative sample in terms of region and the type of the municipality. The size distribution of the companies in the study naturally differed from that of all Finnish companies as those with less than ten employees were excluded. For the same reason of exclusion of small firms, the industry distribution of the included companies differed from that of all Finnish companies. 'Real estate, renting, and business activities' and 'financial intermediation' were underrepresented and 'mining and quarrying plus manufacturing' slightly overrepresented. (See additional file 1 for the size and the industry distribution of the companies.)

The key ratios for economic performance were about the same as for all Finnish firms (including also other firms, not only companies) except for relative indebtedness and quick ratio which were lower in the companies included in the study (table 1). The correlations between key ratios for profitability and solidity were big within a year (absolute values 0.4-0.9) and small for the key ratios for liquidity (0.2). Between the time periods the correlations got smaller.

**Table 1:** Key ratios for economic performance of companies in 2001 (N = 6,271)

**Key ratio**	**Mean**	**Median**	**Standard Deviation**
Profitability			
Operating margin, %	10.0	8.7	8.3
Operating profit, %	6.5	5.7	7.2
Net result, %	4.2	3.6	6.2
Total result, %	4.0	3.3	5.7
Profit/loss for the accounting period, %	4.1	3.2	5.6

Solidity			
Return on capital assets, %	14.0	12.4	13.6
Return on investment, %	26.8	21.5	32.3
Return on equity, %	24.7	21.0	59.9
Relative indebtedness, %	32.2	23.9	28.4
Equity ratio, %	43.4	43.1	23.4

Liquidity			
Quick Ratio	0.51	0.23	0.73
Current Ratio	0.54	0.38	0.61

The average turnover of the companies in the study was about €24 million, and the average age of a company was 18 years.

Of the companies included in the study, 19% had not applied for reimbursement in 2001. Among the companies who had applied for reimbursement, the costs were the highest for the companies' own OHS units and lowest for municipal health centres (table 2). The magnitude of OHS expenditure was relatively small: the total OHS costs represented about 0.1% of turnover on average.

**Table 2:** Companies' costs for occupational health services in 2001 by provider in euro per employee per year

	**Preventive services**		**Curative medical services**	
**Provider**	**Mean**	**Standard deviation**	**Mean**	**Standard deviation**
Employer's own OHS unit (N = 454)	91.60	44.90	138.10	67.15
Employers' joint OHS unit (N = 470)	72.95	37.80	107.30	60.70
Municipal health care centre (N = 1,603)	43.35	35.25	27.10	43.20
Private medical centre (N = 2,422)	62.90	41.55	120.40	78.25
Other (N = 35)	69.60	46.10	125.50	74.90

### Economic performance and applying for reimbursement for the OHS costs

In table 3 the odds ratio for operating margin in 1999 indicates that the relation with OHS is negligible and non-significant after adjustment for the various confounders. The results of the models with other key ratios for economic performance were similar to operating margin and also not significant (data not shown).

**Table 3:** Logistic regression on a company's economic performance in 1999 for a company's probability to apply for reimbursement in 2001 (N = 6,155).

	**Variables in the model**	**OR**	**95% Confidence interval**
Economic Performance Confounders	Operating margin in 1999 (%)	1.00	0.99 to 1.01
	Log turnover in 2001 (€)	2.34	2.16 to 2.53
	Age of company (years)	1.03	1.02 to 1.03
	Share of blue-collar workers in 2001 (%)	0.71	0.50 to 1.01
Industry (categorical)	Reference: Wholesale and retail trade		
	Agriculture, hunting and forestry, fishing	1.33	0.63 to 2.84
	Mining and quarrying, manufacturing	2.32	1.84 to 2.92
	Electricity, gas, and water supply	3.45	1.13 to 10.54
	Construction	1.48	1.16 to 1.89
	Hotels and restaurants	1.65	1.16 to 2.34
	Transport, storage and communication	1.43	1.03 to 1.99
	Real estate, renting, and business	2.89	2.19 to 3.80
	Education	1.47	0.29 to 7.39
	Health and social work	3.14	1.58 to 6.24
	Community, social and personal service activities	2.70	1.50 to 4.84
Region (categorical)	Reference: Uusimaa (region around the capital city)		
	South	1.33	1.12 to 1.58
	East	1.32	1.00 to 1.74
	Central	1.12	0.88 to 1.42
	North	1.07	0.81 to 1.41
	Åland	0.36	0.15 to 0.85
Municipality (categorical)	Reference: Rural		
	Urban	2.26	1.82 to 2.80
	Semi-urban	1.33	1.05 to 1.70

Pseudo R^2 ^= 0.15

### Costs of curative medical and preventive services

Operating profit in 1999 was not related to the costs for curative medical services nor to costs for preventive OHS per employee in 2001 (table 4). The results were similarly non-significant for the other key rations for profitability, solidity and liquidity (data not shown). There were differences between industries, regions and OHS providers.

**Table 4:** Regression models on company's economic performance for costs of curative medical services and prevention (log euros per employee) in 2001 (N = 4,958).

	**Curative medical services, euros per employee (log)**		**Prevention, euros per employee (log)**	
	
**Variables in the model**	**Coefficient**	**95% Confidence interval**	**Coefficient**	**95% Confidence interval**
Constant	0.66	-0.00 to 1.33	2.57	2.22 to 2.93
Operating profit in 1999	-0.001	-0.01 to 0.00	-0.001	-0.00 to 0.00
Age of company	0.00	-0.00 to 0.00	0.00	-0.00 to 0.00
Log turnover in 2001	0.20	0.17 to 0.21	0.10	0.08 to 0.11
Blue-collar workers % in 2001	-0.31	-0.50 to -0.12	0.05	-0.05 to 0.15
Provider model				
Ref. company's own OHS	0		0	
Joint OHS unit	0.02	-0.17 to 0.21	-0.06	-0.16 to 0.04
Municipal OHS	-2.32	-2.49 to -2.15	-0.75	-0.84 to -0.66
Private medical centre	0.02	-0.14 to 0.17	-0.23	-0.31 to -0.14
Other provider	0.18	-0.33 to 0.68	-0.23	-0.50 to 0.04
Industry				
Ref.: Wholesale and retail trade	0		0	
Agriculture, hunting and forestry, fishing	-0.87	-1.44 to -0.31	0.16	-0.14 to 0.46
Mining and quarrying, manufacturing	0.09	-0.04 to 0.22	0.38	0.31 to 0.45
Electricity, gas and water supply	0.32	-0.05 to 0.69	0.38	0.18 to 0.58
Construction	-0.33	-0.48 to -0.18	0.32	0.24 to 0.40
Hotels and restaurants	0.07	-0.17 to 0.31	0.02	-0.11 to 0.15
Transport, storage and communication	-0.18	-0.38 to 0.02	0.02	-0.08 to 0.13
Real estate, renting and business activities	0.36	0.21 to 0.52	0.24	0.16 to 0.32
Education	0.44	-0.55 to 1.44	0.29	-0.24 to 0.83
Health and social work	0.20	-0.18 to 0.59	0.25	0.05 to 0.46
Other community, social and personal service activities	0.49	0.16 to 0.82	0.25	0.08 to 0.43
Ref.: Wholesale and retail trade				
Region				
Reference Uusimaa	0		0	
South	0.18	0.08 to 0.28	-0.11	-0.16 to -0.05
East	-0.14	-0.31 to 0.02	-0.11	-0.20 to -0.03
Central	-0.09	-0.24 to 0.06	-0.18	-0.26 to -0.10
North	-0.05	-0.22 to 0.12	0.20	0.10 to 0.29
Åland	-0.27	-0.89 to 0.35	0.22	-0.11 to 0.55
Municipality				
Reference rural	0		0	
Urban	0.82	0.68 to 0.97	-0.09	-0.16 to -0.01
Semi-urban	0.51	0.35 to 0.68	-0.10	-0.19 to -0.01
Adjusted R^2^	0.46		0.19	
Reset F(3, 4955)	27.28	0.0000	0.47	0.7062

The costs of preventive OHS were higher in companies with higher turnover and for companies in the industries 'manufacturing and mining', 'electricity, gas and water supply', 'construction', 'real estate, renting and business activities', 'health and social work' and 'other community, social and personal service activities' compared to 'wholesale and retail trade'.

## Discussion

The preceding economic performance two years earlier was not statistically related to expenditure on preventive or curative medical OH services in 2001. Economic performance was measured by the annual profitability of a firm, the accumulated wealth that represents the performance of a company through its whole history and liquidity.

The strength of our study was that the data we used was of good quality and covered a vast subgroup representative of Finnish companies. We were the first ones to be able to combine financial statements and OHS data for a company. In addition, we were able to take into account various confounders that affect both the economic performance and the OHS expenditure of a company. Exclusion criteria for continuous business and having more than 10 employees might bias the results into the direction of apparently diminishing the impact of economic performance on a company's OHS expenditure. Entering and exiting companies and companies of small size might be in a more unfavourable financial situation.

Almost one fifth of the companies did not apply for reimbursement although preventive OHS is compulsory for all of them. This could be due to the following reasons. First, the company might not have obeyed the law, and did not organize OHS. This, however, is quite rare as Finnish employers do. The coverage among employees is one of the highest in the world, about 90% [14]. Only small enterprises with less than 20 employees do not always have a contract with a provider. Secondly, in small companies, it is possible to have years with no need for OHS activities, and therefore without costs. And sometimes companies just do not apply for reimbursement, as the costs of OHS might be low and filling in the application might constitute a more significant expenditure.

Our results differ from those in safety studies [4,9,15] where a firms' investment in safety is affected by economic success, at least in firms performing most poorly. A difference with safety measures is that OHS are of a more stable nature. Employees continue to use the services and providers continue to provide the services apparently irrespective of a company's economic performance. In addition, in occupational health services, like in general in health care, information asymmetry exists between the provider and both the payer (employer) and the user (employee). Therefore, the employer has to use the expertise of the provider in deciding upon the services. This leaves less space for the company to decide on the contents and the costs of OHS. Moreover, the total costs are about 0.1% of a company's turnover. This means that the expenditure on OHS has only a minor impact on a company's finances. This was also confirmed in our article based on the same data [16]. Company's investment in preventive OHS did not have a positive impact on company's economic performance.

Although the economic performance of a company did not affect the amount of money spent per employee in curative medical services and prevention in a particular company, there were differences between regions, industries and OHS providers. The OHS system is not entirely successful in optimal allocation of resources according to needs. Expenditure on prevention is not the highest in the riskiest industries [17,18] and white-collar workers benefit more in terms of free use of curative medical services [19]. In addition, regional differences are connected to the supply of the OHS services.

## Conclusion

Expenditure on OHS seems to be independent of a company's economic performance in Finland. Legislation obligating the employers and the reimbursement system both contribute to this.

## Competing interests

The authors declare that they have no competing interests.

## Authors' contributions

EK and HV planned the study. AK carried out the analysis under supervision by HV and EK. EK drafted the manuscript, and the other authors have read and approved it.

## Appendix 1

Definitions for key ratios of economic performance 5.

**Table 5:** Definitions for key ratios of economic performance

Profitability	
	
Operating margin %	Company's earnings that is left over after paying for variable costs of production divided by net sales.
Operating profit %	Earnings before interest and taxes (EBIT) divided by net sales.
Net result %	(Total revenues -- total expenses) divided by net sales = tells if a company has earned or lost money in an accounting period with its business.
Total result %	Net result + extraordinary incomes -- extraordinary expenses divided by net sales
Profit/loss for the accounting period %	
	The profit/loss result after the company has paid the taxes divided by net sales.
	
Solidity	
	
Return on Capital Assets %	Tells how profitable the company is relative to its total assets. = Net income/total assets
Return on investment %	Evaluates the efficiency of an investment = (gain from investment -- cost of investment)/cost of investment.
Return on equity %	Tells how much profit is made relative to the owners investment in the company = Net income/shareholders equity
Relative indebtedness %	Company's liabilities divided by its turnover. Less than 40%: Good 40--80%: Satisfactory More than 80%: Poor
Equity ratio %	The percentage of equities from the balance sheet Over 40%: Good 20-40%: Satisfactory Less than 20%: Poor
Liquidity	
	
Quick ratio	Company's ability to meet its obligations. Over 1: Good 0.5-1: Satisfactory Less than 0.5: Poor
Current ratio	Company's ability to meet short term debt obligations. Over 2: Good 1-2: Satisfactory less than 1 poor

## Pre-publication history

The pre-publication history for this paper can be accessed here:



## Supplementary Material

Additional file 1 **Companies in the study by number of employees and industry in 2001**. The size and industry distribution (number of firms, %) of the companies included in the study. Click here for file
