# Supplementary material for: Does the company's economic performance affect access to occupational health services?
Source: BMC Health Serv Res. 2009 Sep 2;9:156. doi: 10.1186/1472-6963-9-156 (PMC2753325; doi:10.1186/1472-6963-9-156)
Supplement: Additional file 1 — Companies in the study by number of employees and industry in 2001. The size and industry distribution (number of firms, %) of the companies included in the study. [file 1472-6963-9-156-S1.doc]

**Additional file 1 Companies in the study by number of employees and industry in 2001**

| Number of companies  (per cent by row)  [per cent by column] | Industry | | | | | | | | | | | |
| --- | --- | --- | --- | --- | --- | --- | --- | --- | --- | --- | --- | --- |
| Number of employees | AB | CD | E | F | G | H | I | K | M | N | O | Total |
| 10-19 | 32  (1.5)  [66.7] | 554  (25.8)  [24.0] | 16  (0.7)  [21.3] | 375  (17.4)  [42.5] | 523  (24.3)  [38.5] | 79  (3.7)  [34.5] | 199  (9.3)  [44.2] | 272  (12.7)  [38.2] | 3  (0.1)  [21.4] | 54  (2.5)  [61.4] | 44  (2.1)  [42.7] | 2151  [34.3] |
| 20-99 | 15  (0.5)  [31.3] | 1287  (40.3)  [55.7] | 48  (1.5)  [64.0] | 437  (13.7)  [49.5] | 687  (21.5)  [50.6] | 125  (3.9)  [54.6] | 175  (5.5)  [38.9] | 339  (10.6)  [47.6] | 9  (0.3)  [64.3] | 25  (0.8)  [28.4] | 45  (1.4)  [43.7] | 3192  [50.9] |
| 100-499 | 1  (0.1)  [2.1] | 370  (49.8)  [16.0] | 10  (1.4)  [13.3] | 59  (7.9)  [6.7] | 117  (15.8)  [8.6] | 21  (2.8)  [9.2] | 61  (8.2)  [13.6] | 88  (11.8)  [12.3] | 1  (0.1)  [7.1] | 5  (0.7)  [5.7] | 10  (1.4)  [9.7] | 743  [11.8] |
| 500- | 0  (0)  [0] | 98  (53.0)  [4.2] | 1  (0.5)  [1.3] | 12  (6.5)  [1.4] | 32  (17.3)  [2.4] | 4  (2.2)  [1.8] | 15  (8.1)  [3.3] | 14  (7.6)  [2.0] | 1  (0.5)  [7.1] | 4  (2.2)  [4.6] | 4  (2.2)  [3.9] | 185  [3.0] |
| Total | 48  (0.8) | 2309  (36.8) | 75  (1.2) | 883  (14.1) | 1359  (21.7) | 229  (3.7) | 450  (7.2) | 713  (11.4) | 14  (0.2) | 88  (1.4) | 103  (1.6) | 6271  (100) |

AB Agriculture, hunting and forestry, fishing

CD Mining and quarrying, manufacturing

E Electricity, gas, and water supply

F Construction

G Wholesale and retail trade

H Hotels and restaurants

I Transport, storage, and communication

K Real estate, renting, and business activities

L Public administration and defence; compulsory social security

M Education

N Health and social work

O Other community, social, and personal service activities
